# Genetical Genomics of Tonic Immobility in the Chicken

**DOI:** 10.3390/genes10050341

**Published:** 2019-05-07

**Authors:** Jesper Fogelholm, Samuel Inkabi, Andrey Höglund, Robin Abbey-Lee, Martin Johnsson, Per Jensen, Rie Henriksen, Dominic Wright

**Affiliations:** 1 AVIAN Behavioural Genomics and Physiology Group, IFM Biology, Linköping University, 58183 Linköping, Sweden; jesper.fogelholm@liu.se (J.F.); samin711@student.liu.se (S.I.); andrey.hoglund@liu.se (A.H.); robin.abbey-lee@liu.se (R.A.-L.); per.jensen@liu.se (P.J.); rie.henriksen@liu.se (R.H.); 2 The Roslin Institute and Royal (Dick) School of Veterinary Studies, The University of Edinburgh, Midlothian, Scotland EH25 9RG, UK; sorill@gmail.com; 3 Department of Animal Breeding and Genetics, Swedish University of Agricultural Sciences, Box 7023, 750 07 Uppsala, Sweden

**Keywords:** QTL, eQTL, tonic immobility, behaviour, domestication, muscle pH

## Abstract

Identifying the molecular mechanisms of animal behaviour is an enduring goal for researchers. Gaining insight into these mechanisms enables us to gain a greater understanding of behaviour and their genetic control. In this paper, we perform Quantitative Trait Loci (QTL) mapping of tonic immobility behaviour in an advanced intercross line between wild and domestic chickens. Genes located within the QTL interval were further investigated using global expression QTL (eQTL) mapping from hypothalamus tissue, as well as causality analysis. This identified five candidate genes, with the genes *PRDX4* and *ACOT9* emerging as the best supported candidates. In addition, we also investigated the connection between tonic immobility, meat pH and struggling behaviour, as the two candidate genes *PRDX4* and *ACOT9* have previously been implicated in controlling muscle pH at slaughter. We did not find any phenotypic correlations between tonic immobility, struggling behaviour and muscle pH in a smaller additional cohort, despite these behaviours being repeatable within-test.

## 1. Introduction

Identifying the molecular mechanisms of animal behaviour is an enduring goal for researchers. Gaining insight into these mechanisms enables us to gain a greater understanding of behaviour in domestic animals, as well as their wild counterparts. Understanding the genetic determinants of behaviour in domestic populations can also improve animal health and welfare, as well as productivity in production environments. In this paper, we study the genetic determinants of tonic immobility, a freezing behaviour, and the phenotypic relationship between tonic immobility, meat pH and struggling behaviour in a population of wild x domestic intercross chickens. 

Tonic immobility, originally considered a form of animal hypnosis, is one of the classical behaviours that have been studied by both ethologists and behavioural geneticists alike, and was first described in the mid-1600s [1]. It is believed to be an anti-predator behaviour, that will only manifest in a natural setting if a prey animal has exhausted all other possible means of escape from a predator [2], though the behaviour is so widespread in such a variety of species that it has also been thought of as a reflex response, cerebral inhibition or even paralysis through fear [3]. As an anti-predation strategy, when the prey animal has been caught by the predator it will freeze and remain motionless for a considerable amount of time. This can deceive the predator into believing that the prey animal has been subjugated, which will have the effect that the predator starts to search for another prey item which will allow the caught prey to escape its predator. In this way, the behaviour can potentially increase survival chances by 50% [4]. 

Tonic immobility has been studied in a wide variety of species such as blue crabs [5], quail [6] as well as chickens [7], and even sharks [8]. Animals that are subjected to this test in a laboratory setting are placed on their back and gently restrained for a brief period. The longer the animal stays in this immobile state, the more fearful it is considered to be [9], though the relationship between tonic immobility and anxiety is potentially less straight forward, as it often does not correlate with other basic anxiety measures [10,11]. However, numerous experiments have been made that demonstrate that the duration of tonic immobility will be prolonged by making the situation appear more threatening to the animal, or subjecting it to aversive stimuli just before the tonic immobility trial [9,12]. Another study demonstrated that chicks that had been brought up with a broody hen had a significantly shorter duration of tonic immobility compared to chicks which had been raised alone, which could indicate that they had a reduced fear level [13]. 

The genetic basis of tonic immobility has been investigated using both heritability and linkage assays. Initial selection experiments in chickens [14] and quails [15] gave heritability estimates between 50–90%. Quantitative trait loci (QTL) studies on tonic immobility have been performed in a wild x domestic chicken intercross [10,11] and an intercross between selected quail lines for high and low tonic immobility time [16,17], with both studies identifying loci on chromosome 1. Despite this behaviour being apparent in such a wide range of species, as of now no causal genes have yet been identified. Given that clear evidence that this behaviour is truly adaptive, or what precisely it is adaptive for, is still lacking, there remains the possibility that identifying the genetic determinants underpinning this behaviour can help elucidate its precise role. 

As well as a putatively adaptive role, tonic immobility could also impact on more direct welfare and production-related traits, with several potential production costs associated with tonic immobility behaviour in poultry farming and industry settings. In particular, time spent in tonic immobility has been correlated with behavioural and stress responses during shackling [18], with birds shackled for longer periods having a greater tonic immobility duration [18]. Shackling occurs immediately before slaughter, when birds are restrained and then inverted on the shackle line, before being stunned and then decapitated [18,19]. This is a highly stressful time for the birds, with shackling time increasing corticosterone production [19]. Birds are required by EU law to be sufficiently relaxed to allow stunning to be carried out effectively (Council Directive 93/119/EC); however there is no set maximum time lapse between shackling and stunning, meaning birds that flap for extended periods will remain on the line for longer. The amount of movement and the degree of struggling during this phase will influence meat quality [20]. Struggling and excessive movement on the transportation line will lead to a build-up of lactic acid in the muscle, this decreases the pH and hence the water holding capacity of the muscle, leading to drier and less palatable meat [20]. This meat quality problem is referred to as pale soft exudative (PSE) meat and typically occurs in 20–48% of poultry production [21,22,23]. Given that 90 million tons of chicken meat are produced annually, and that up to 48% of meat markets may be affected, this massively affects sustainability and production costs. Despite the economic and welfare issues surrounding this trait, very little is known about the underlying genetics. Identification of causal and selectable genetic loci would, therefore, have a huge cost-saving potential. One study to map such traits was performed by Nadaf et al. [24] and identified five QTL for muscle pH 15 min post-slaughter in a high x low growth chicken intercross. The high growth birds, in this case, moved more and performed significantly more “straightening up” events, while correlations between wing flapping and pH were also detected. Gene expression assays in the candidate region identified expression QTL (eQTL) for four genes in the candidate QTL region— *PRDX4, ACOT9, KLHL15* and *APOO* [25]. Targeted resequencing of the QTL region corroborated the previous results and highlighted *PRDX4* and *ACOT9* as likely candidate genes [26]. In addition, *PRDX4* and *ACOT9* were both present in a candidate QTL interval for tonic immobility in the quail selected lines [27].

We have previously identified strong candidate genes for anxiety and sociality behaviour by using a combined QTL and eQTL approach in an advanced intercross between wild and domestic chickens [28,29,30,31]. Although the identification of the genes underlying behaviour is always problematic, this approach has proven to be very powerful by combining expression profiles for over one hundred individuals with simultaneous genotyping and phenotyping, to allow the assessment of the relationship between genotype, gene expression and phenotype. One of the main limitations of a standard F_2_ linkage intercross is resolution, with relatively large areas identified. By using an advanced intercross, we have generated far more recombination events, which when combined with the naturally high recombination rate in the chicken [32], gives much narrower confidence intervals than is standard [33]. We use a three-stage approach, whereby first behavioural QTL and expression QTL from hypothalamus tissue are mapped in the same intercross. In the next stage, all eQTL that overlap behavioural QTL are then considered as putative candidates for further examination. In the third stage, the expression level for each putative eQTL is then correlated with the behavioural trait for the relevant overlapping QTL, with any that are significant then retained and finally causal gene analysis acts as the final statistical test. 

In this study, a whole genome linkage scan was performed in an eighth generation advanced intercross between wild and domestic chickens to identify QTL affecting tonic immobility behaviour. These QTL were then combined with an eQTL scan conducted previously on the same individuals using hypothalamus tissue, to identify putatively causal genes affecting tonic immobility behaviour in the chicken. In addition to this, the link between tonic immobility, struggling behaviour and breast muscle pH was explored in a further generation of the advanced intercross, a subset of F_13_ Individuals (*n* = 44), by measuring the correlation between tonic immobility, struggling behaviour and meat pH. 

## 2. Methods 

### 2.1. Primary Genetical Genomics Mapping Study

#### 2.1.1. Chicken Study Population and Cross Design

The animals used for the main combined QTL/eQTL mapping study were generated from an intercross population between a line of selected White Leghorn chickens maintained from the 1960s and a population of Red Junglefowl originally from Thailand [10,34], that is maintained as an advanced intercross population. One male Red Junglefowl and three female White Leghorn were used to found the intercross and generate 41 F_1_ progeny. The intercross has been maintained at a population size of approximately 100 birds per generation until the F_7_ generation. A 50:50 sex ratio was maintained in each generation, with all individuals used to create the subsequent generation. The intercross has been used to identify QTL for a number of different behavioural, morphological and life history traits [11,29,31,34,35,36,37,38,39,40,41]. A total of 532 of the F_8_ individuals generated were assayed for tonic immobility behaviour. Of these individuals, 129 of the F_8_ were used in the eQTL experiment, using hypothalamus/ thalamus tissue dissected out at 212 days of age, and run individually on a 135k probe microarray (see [42]). For further details on feed and housing see [28]. 

#### 2.1.2. Choice of Hypothalamus Tissue

The selection of tissue that potentially affects tonic immobility behaviour is fairly problematic, given the lack of knowledge as to the genetic basis of the behaviour. Tonic immobility has been most strongly linked with stress/anxiety/anti-predation behaviour, so we therefore, selected hypothalamus tissue due to its pivotal role in the hypothalamic–pituitary–adrenal (HPA) axis (integral to stress and anxiety responses). It is known for its effects on anxiety-related behaviour [43,44,45], its control of the amygdala [44] (also highly involved in the control of anxiety) and its direct role in regulating tonic immobility behaviour [46]. The control of the amygdala is important as the central nucleus of the amygdala has also been found to affect tonic immobility behaviour [47]. Of course, an important caveat is that candidate genes may well be missed due to the incorrect tissue type being selected, though candidate genes that are identified will still be valid.

#### 2.1.3. F_8_ Phenotyping

Tonic immobility was assessed in both test generations (F_8_ and F_13_). Tests were carried out when the animals were fully grown and sexually mature (~170 days for the F_8_ generation, ~104 weeks for the F_13_ generation). Tests were performed in a nearby room to the home pen, with birds carried there individually immediately before the tonic immobility test to minimise stress. Birds were inverted and placed on their back in a V-shaped wooden cradle (approximately 50 cm in length), with gentle manual pressure applied to the chest of the animal for 10 s before slowly releasing the bird. If the bird righted itself within 30 s another attempt was made to put the bird in tonic immobility, allowing up to three attempts per bird. If the bird went into tonic immobility the duration was measured, and the maximum time recorded was capped to 600 s (so individuals that would remain in tonic immobility for periods longer than 600 s instead received a score of 600 s). Tonic immobility duration was considered from when the bird was initially placed upside down until the bird was fully righted. Each individual was tested in the tonic immobility trial twice with at least one week between trials. The phenotypes that were derived from these trials was time spent in tonic immobility in trial 1, time spent in tonic immobility in trial 2, average time spent in tonic immobility over the two trials, and maximum time spent in tonic immobility (the highest recorded score from either trial 1 or trial 2). Descriptive statistics and correlations between the different tonic immobility measures are presented in Supplementary Tables S1 and S2.

#### 2.1.4. Genotyping, QTL and eQTL Mapping

DNA extraction was performed on blood using a standard salt extraction protocol by Agowa GmbH (Berlin, Germany). A total of 652 SNP markers (551 of which were fully informative) were genotyped per individual, and used to calculate a linkage map of length ~9267.5 cM, with an average marker spacing of ~16 cM (see [29] for a full list of markers). Markers were genotyped using an Illumina GoldenGate assay (see [29] for further details). The R/qtl software package [48] was used to perform the QTL analysis, with both standard interval mapping and epistatic analyses performed. Interval mapping was calculated using both additive and additive plus dominance models. For the tonic immobility QTL analysis in the F_8_ individuals, batch, sex, and weight at 212 days were always included as covariates. A principal component analysis (PCA) of the first ten principal components of the genotypic data were fitted to account for population substructure (see [42] for details), with all significant principal components retained in the final model. A sex-interaction effect was added, where significant, to account for a particular QTL varying between the sexes. Digenic epistatic analysis was performed as per the guidelines given in [49]. A global model was constructed for each trait that incorporated standard main effects, sex interactions and epistasis. The most significant loci were added to the model first, followed by the less significant loci. 

eQTL mapping was also performed on hypothalamus tissue in the individuals on the F_8_ individuals cross using R/qtl [48], as has been documented previously [42]. In brief, both cis and trans eQTL were detected. The assay itself was based on a Roche Nimblegen custom 135k probe array that incorporated all known Ensembl genes, plus ESTs were taken from an EST library of the chicken brain. See [42] for further details of the methods and analyses performed. In the case of local, potentially cis-acting, eQTL (defined as a QTL located in close proximity to the target gene affected), one was called if a signal was detected in the closest flanking markers to the gene in question, to a minimum of 100 cM around the gene (i.e., 50cM upstream and downstream of the gene). The distance of 50 cM was used to ensure that at least two markers up and down-stream from the gene location were selected to enable interval mapping to be performed. The trans-eQTL scan was performed on the whole genome, rather than being limited to the gene location, with a genome-wide empirical significance threshold generated. In total 535 local eQTL and 99 trans eQTL were identified [42]. One note here is that the build used for the eQTL analysis in [42] was Galgal4, which may mean that some cis eQTL have been missed when the gene was incorrectly placed on the genome, relative to the SNP marker map that they were mapped against. In the case of trans eQTL, the potential movement of genes is not an issue, as the whole genome is being scanned.

#### 2.1.5. Significance Thresholds

Significance thresholds for the QTL analysis were calculated by permutation [50,51]. We used a genome-wide 20% threshold as suggestive (with this a slightly more conservative threshold than the standard suggestive threshold [52]), with a 5% genome-wide level being significant. The 5% significant threshold was LOD ~4.4, while the suggestive threshold was ~3.6 (thresholds were calculated individually for each phenotype). Four different traits were mapped, and although strongly correlated we also applied a multiple testing correction. As LOD scores are based on a log10 scale, a multiple testing correction of 4 = 0.6 increase in LOD. This led to a significant threshold of ~5.0 and a suggestive threshold of ~4.0. Confidence intervals for each QTL were calculated using a 1.8 LOD drop method (i.e., where the LOD score on either side of the peak decreases by 1.8 LOD), with this being equivalent to a 95% confidence interval for an intercross type population [53]. The nearest marker to this 1.8 LOD decrease was then used to give the confidence intervals in megabases. Epistatic interactions were also assessed using a permutation threshold generated using R/qtl, with a 20% suggestive and 5% significant genome-wide threshold once again used. In the case of epistatic loci, the approximate average LOD significance threshold for pairs of loci were as follows (using the guidelines given in [49]): full model ~11, full versus one ~9, interactive ~7, additive ~7, additive versus one ~4. 

#### 2.1.6. Analysis of Candidate Genes in the F_8_ Generation (eQTL Genes Falling within QTL Intervals)

Significant tonic immobility QTL were overlapped with the previously identified eQTL, and all significant eQTL genes were then tested as candidate genes for the tonic immobility QTL with which they overlapped. These candidate genes were then modelled using the gene expression value on the behavioural trait for the QTL of interest, i.e., if an eQTL overlapped a QTL for time spent in tonic immobility, the eQTL gene expression trait would be correlated with this phenotype. The linear model for this analysis used the behavioural trait as the response variable and the expression trait as the predictor and included sex and batch as factors. The *p*-values for the regression coefficient were Bonferroni corrected for the number of uncorrelated eQTL in the QTL region. eQTL that were present within a QTL confidence interval, which were also significantly correlated with the QTL trait, were then considered to be candidates. Network edge orientation (NEO) analysis was then used to assess causality further (see below). One issue with this approach is that the behavioural QTL were based on up to 532 individuals, whereas the eQTL/expression phenotypes were only available for 129 individuals. Therefore, causality testing was only applied where the behavioural QTL was detectable in the smaller dataset (*n* = 129).

To attempt to ascertain if a gene that has been identified as being correlated with a given phenotype is causal, it is possible to perform a causality analysis. In this case, by using the SNP marker that underlies both the eQTL and behavioural QTL traits, and then by looking at the respective correlations between the eQTL and behavioural QTL and their correlations with one another it is possible to obtain statistical significance for causality (i.e., that a given gene is causal to a particular phenotypic QTL). Causality analysis in the study here consisted of structural equation modelling with the network edge orientation (NEO) software [54]. This software uses both single SNP marker and two SNP marker analyses, comparing a causal model (where the genotype affects behaviour using changing gene expression) to four alternative models, reflecting other possibilities:

- CAUSAL: Genotype modifies gene expression which in turn modifies behaviour (genotype -> expression trait -> behaviour).
- REACTIVE: Genotype modifies behaviour which in turn modifies the expression trait (genotype -> behaviour -> expression trait)
- CONFOUNDED: Genotype modifies both the expression trait and the behaviour separately (expression trait <- genotype -> behaviour)
- COLLIDER B (behaviour is the collider): Genotype and the expression trait both independently modify behaviour (genotype -> behaviour <- expression trait)
- COLLIDER E: (expression is the collider): Genotype and behaviour both independently modify the expression trait (genotype -> expression trait <- behaviour).

The NEO software uses expression traits and phenotypic traits described in undirected trait networks. Causal information is then encoded by directed networks when A causes B if trait A causally influences trait B, with the process of assigning a causal direction to some of the edges in a network known as “edge orienting” [54]. By also including an SNP “anchor” (the orientation of the marker to trait A being unambiguous), it is possible to use the partial correlation coefficients to generate a local edge orientation (leo.nb) score. This is used to study the fit of the causal models, with a high score indicating a better fit (i.e., a model that is causal). In addition, the fit of each single marker model is also tested using a chi-squared test with 1 degree of freedom, with NEO referring to this as the model *p*-value. With this model *p*-value, “the main point is that the higher the model *p*-value, the better the causal model fits the data”, to quote Aten et al. [54], i.e., if this model *p*-value is non-significant (*p* > 0.05), this indicates that only the causal model fits the data (other models are rejected). A significant model *p*-value (*p* < 0.05) despite a high leo.nb score would mean that it is not possible to distinguish between the different models fitting the data (i.e., multiple models fit). The analysis, therefore, generates two measures—the local edge orientating score, and a *p*-value that compares the best fitting model with the next best fitting model. Aten et al. [54] recommend using the leo.nb.cpa score when a marker affects both A and B traits, and recommend significance is called when this score is > 1.0. More recently, several studies have used a leo.nb.cpa score of 0.3 as a suggestive level of significance [55,56]. When two different markers underlie each of the traits, they recommend using the leo.nb.oca score, and use a threshold of >0.3 for significance. In addition to this, NEO suggests users inspect the *p*-value of the causal model to make sure the fit is good. If this *p*-value is non-significant (*p* > 0.05), this indicates that only the causal model fits the data (other models are rejected). A significant *p*-value (*p* < 0.05) despite a high leo.nb score would mean that none of the models fit the data very well. For each gene, we report the leo.nb score and the *p*-value of the causal model. 

### 2.2. Verification Cohort and the Relationship between Tonic Immobility, Inverted Restraint and pH

To assess the relationship between tonic immobility behaviour, shackling behaviour and breast muscle pH at slaughter, we used a further generation (F_13_) of the advanced intercross. The subsequent generations after the F_8_ generation were maintained at ~100 individuals per generation, until the F_13_ generation. In the F_13_ generation, 44 individuals were measured for tonic immobility, the inverted restraint test (a proxy of struggling behaviour on a shackle line), and breast muscle pH at slaughter.

#### 2.2.1. Tonic Immobility

Tonic immobility was recorded in the F_13_ cohort in the same manner as in the F_8_ cohort (see above). Two trials per bird were once again performed.

#### 2.2.2. Inverted Restraint Test 

In the case of the F_13_ cohort, to mimic the struggling behaviour that occurs during placement on the shackle line, a second behavioural test measured the degree of struggling and the duration of self-righting attempts each bird performed when held upside down. This test was termed an inverted restrain test and was carried out after the tonic immobility trials. The test was performed by securing birds around the ankles with one hand before inverting them at a height of ~1 m. Each individual was tested twice by the same experimenter, with at least a week between any two behavioural tests. The test was recorded with a digital video camera (Canon Legria HF M52, Tokyo, Japan), and the number of wing flaps performed scored, as well as the duration of self-righting behaviour in seconds. This test was used as a comparison between tonic immobility behaviour and inverted restraint behaviour. The second test was performed immediately before slaughter (termed the pre-slaughter inverted restraint test), and the measurement was used to correlate the degree of struggle during the inverted restraint with breast muscle pH post-slaughter. Birds were ~106 weeks of age for the final test.

#### 2.2.3. pH Measurements

Birds were culled via cervical neck dislocation and decapitation at an age of ~106 weeks. Once birds were culled, 15 min post mortem, an incision was made in the right breast muscle and a HI 99121 (Hanna Instruments) pH probe was used to measure the pH for 5 min. After this procedure, the entire breast muscle was dissected out and stored on ice in preparation for the second pH measurement which took place ~5–6 h post mortem. 

#### 2.2.4. Statistical Analysis

We estimated pairwise correlations between tonic immobility, inverted restraint test behaviour and breast muscle pH in the F_13_ cohort. We used partial Spearman’s rank correlations controlling for sex, using the R package ppcor [57] to perform the partial correlations comparing the different behavioural and pH trait measurements. 

### 2.3. Data Availability 

Microarray data for the chicken hypothalamus tissue are available at E-MTAB-3154 in ArrayExpress.

### 2.4. Ethics Statement

The study was approved by the local Ethical Committee of the Swedish National Board for Laboratory Animals, Ethical permit Dnr 50-13.

## 3. Results

### 3.1. Genetic Architecture of Tonic Immobility

In total, we detected 18 genome-wide significant QTL regions on 10 different chromosomes that affect various aspects of tonic immobility behaviour. Several of these overlapped, resulting in 15 discrete tonic immobility QTL in total. Of these, 12 of the 18 QTL had greater effects in the Red Junglefowl (wild) genotype (animals stayed in tonic immobility for a longer duration with the RJF genotype), with three of the QTL that had a greater effect in White Leghorn genotype possessing a dominant rather than additive effect. This appears to confirm that wild genotypes spend longer in tonic immobility as compared to the domestic genotypes. The confidence intervals for the detected QTL were as expected considerably smaller than a standard F_2_ intercross, with the average size for the tonic immobility QTL being ~3.5 Mb. Details for the QTL results can be found in Table 1. 

### 3.2. eQTL Overlap and Candidate Gene Detection

A total of 98 significant eQTL overlapped with tonic immobility QTL, of which seven were significantly correlated with their corresponding tonic immobility behavioural trait (representing five different genes): *ACOT9, PRDX4, PDE7A*, an Expressed Sequence Tag (EST) mapping to within 1kb of *CA8*, and an EST mapping to *VPS26* (see Table 2). Of these, *PRDX4* had the strongest correlation with tonic immobility behaviour (*p* = 0.0002), whilst *ACOT9* was also strongly correlated (*p* = 0.004), as was *VPS26* (*p* = 0.004), see Table 2. Of the five genes, one (*VPS26*) did not have a significant behavioural QTL in the reduced eQTL dataset and so could not be tested using NEO. Although none of the remaining four genes were fully supported by the NEO causal modelling, *PRDX4* and *ACOT9* had the highest scores for maximum time in tonic immobility, with leo.nb.cpa values of 0.38 and 0.55 (see Table 2), with these passing the threshold for suggestivity but not significance. In addition, the other competing gene models (collider, etc.) were all non-significant (*p*-values > 0.90), implying the causal model had the strongest fit. One problem with the NEO analysis for these genes affecting “maximum time in tonic immobility” is that this QTL involved an epistatic interaction with another locus on chromosome 12. When this interaction is not added to the model in the QTL analysis in the reduced eQTL dataset, only the chromosome 12 locus is significant (LOD 1.5), while when the interaction is added both loci are strongly significant (LOD = 5.9 and 6.6). As the interaction term cannot be added to the NEO causality analysis, we instead tested using firstly the chromosome 10 locus as affecting the behavioural QTL, and secondly the chromosome 12 locus as affecting behaviour (see Table 2, with both results given in the table, also Supplementary Table S3). In both cases, the NEO results did not support a causal role, however the failure to incorporate this interaction may potentially explain this non-significance, given the behaviours were very strongly correlated with gene expression (*p* = 0.004 and 0.0002). Both *PRDX4* and *ACOT9* are affected by trans effect eQTL. In addition to *PRDX4* and *ACOT9*, the probe for the gene *CA8* also showed some trend towards causality (leo.nb.cpa of 0.49) for average time spent in tonic immobility, though there was less support for this gene affecting maximum time spent in tonic immobility (leo.nb.cpa 0.31). The remaining genes had low scores. 

### 3.3. Relationship between Tonic Immobility, Inverted Restraint and Breast Muscle pH 

The relationship between tonic immobility, inverted restraint behaviour and breast muscle pH was investigated in a further F_13_ generation of the intercross. In particular, we wished to assess the potential role between freezing due to anti-predator related behaviour (tonic immobility) and struggling on a shackle line that can lead to decreased muscle pH. The two strongest candidate genes we find for tonic immobility (*PRDX4* and *ACOT9*) have also been implicated as regulators of breast muscle pH at slaughter, due to their role in struggling behaviour [25]. The F_13_ individuals were tested for tonic immobility and inverted restraint behaviour, and breast muscle pH at slaughter was measured. The assay repeatability of the tonic immobility test was significant (correlation: 0.43, *p* < 0.01), whilst with both forms of the inverted restraint test, number of flaps performed correlated with the duration of struggle (IIRT correlation: 0.98, *p* < 0.001, PSRT correlation 0.94, *p* < 0.001) through the initial and pre-slaughter inverted restraint tests did not correlate with one another (correlation duration 0.15, *p*-value = 0.33, see Table 3). Breast muscle pH was significantly correlated between measures taken immediately at slaughter and after 6 h (correlation = 0.42, *p* < 0.005). Despite being strongly repeatable, we found no significant correlations between tonic immobility duration and breast muscle pH, or between tonic immobility and inverted restraint tests in the F_13_ cohort (all *p*-values > 0.25, see Table 3). 

## 4. Discussion

Using an advanced intercross between wild and domestic chickens, in combination with a QTL and eQTL approach, we have identified several strong candidate genes affecting tonic immobility behaviour. Two of the genes, *PRDX4* and *ACOT9* have been implicated in muscle pH at slaughter by a previous study [25]. However, in an additional cohort, we find that tonic immobility behaviour appears to be unrelated to struggle during shackling or muscle pH at slaughter. We, therefore, identify novel candidates for tonic immobility, but fail to demonstrate a correlation between tonic immobility and struggle during the shackling line. 

We have previously shown that the use of a combined QTL and eQTL approach can yield high-quality candidate genes that are causal to phenotypes [28,29,30,42]. Of the candidate genes identified for tonic immobility, *PRDX4* and *ACOT9* were the strongest candidates in terms of the strength of correlation between gene expression and behaviour, and also of the causality analysis using NEO. *PRDX4* is involved in neurogenesis, regulation of *HIF1-alpha*, and has previous links to anxiety and sociality behaviour. In the case of previous behavioural effects, these are present both within the same intercross, where we find *PRDX4* as a candidate for open field and social reinstatement behaviour [30,42] as well as in a different species, with pigs shown to have social behaviour correlated with *PRDX4* expression [58]. Given these previous links with anxiety behaviour, *PRDX4* is an excellent candidate for tonic immobility behaviour both in the chicken and in other vertebrates. *ACOT9* was also a strong candidate for tonic immobility behaviour, though fewer behavioural effects have been observed previously for this gene. *ACOT9* is likely an enzyme involved in hydrolysing acyl-coenzyme A thioesters, with the loss of function of acyl-CoA thioesterases potentially involved in neuronal degradation [59]. Experiments performed in mice have shown that *ACOT9* is active in mitochondria where it can catabolise amino acids into propionyl CoA which is subsequently used to produce succinyl CoA that can enter the KREBS cycle [60], thus providing a theoretical link between *ACOT9* and muscle pH. The strength of the correlation between gene expression and tonic immobility behaviour was weaker than for *PRDX4*; however, the causality analysis was slightly stronger in favour of this gene (though both were non-significant by the stringent causality analyses thresholds, they passed the suggestive threshold).

A relationship between tonic immobility and struggling behaviour seems plausible, especially given the role of *PRDX4* on muscle pH at slaughter demonstrated previously by [26]. However, in our exploration of this link in a further generation of the advanced intercross, there was a lack of any correlation between tonic immobility behaviour and inverted restraint behaviour, and tonic immobility behaviour and muscle pH at slaughter. This suggests that either the relationship between tonic immobility and struggling during shackling appear to be more complex than they would first appear, the two are unrelated, or our phenotypic assay is a relatively poor mimic of shackling behaviour. Therefore if *PRDX4* has a pleiotropic effect on breast muscle pH as well as tonic immobility, this comes through different mechanisms rather than solely behavioural effects. The study by Li et al. [25] found correlations between *PRDX4* and muscle pH in muscle tissue, whereas our study focused on the hypothalamus, suggesting a tissue-specific role. To further elucidate this, a full genomic scan for the inverted restraint test would help to identify whether tonic immobility and struggling behaviour share a common genetic architecture. Similarly, a full scan for breast muscle pH in the advanced intercross could help identify whether any alleles for variation in this physiological response are segregating in our particular advanced intercross. 

Both *PRDX4* and *ACOT9* were also identified as being strong candidates for social behaviour in the same F_8_ intercross [30]. Gene expression was associated with time spent in the start zone of the social reinstatement arena (*PRDX4 p* = 0.02, *ACOT9 p* = 0.005), while NEO causality analysis supported these genes affecting this trait [30]. The social reinstatement test (where birds are timed to see how long they seek conspecifics in a novel arena) is considered a blend of anxiety-related traits and sociality-related traits [61]. Similar to the relationship between tonic immobility and inverted restraint behaviour, there is no phenotypic correlation between tonic immobility and social reinstatement measures in these birds (see Supplementary Table S1), yet the genetic architectures and indeed candidate genes appear to overlap. This helps give us an insight into tonic immobility behaviour in general—specifically how a gene can affect social, anxiety and tonic immobility traits, though apparently by different mechanisms. This pleiotropy adds weight to the hypothesis that it is an anxiety/predation-related behaviour, as well as more directly to the actual gene mechanisms that regulate this behaviour.

In addition to *PRDX4* and *ACOT9*, three other genes were identified as candidates for tonic immobility behaviour—*PDE7A*, an EST 1kb from *CA8*, and *VPS26.* The genes *PDE7A* and *VPS26* were significantly correlated with tonic immobility, however, the NEO causality analysis did not support them. Of the three genes, two have been previously linked with behavioural effects (*PDE7A* and *CA8*). *PDE7A* is a cyclic nucleotide phosphodiesterase, and is involved in the regulation of inflammation. It has been linked with cognitive decline [62,63], neurological and inflammatory disorders [64]. During immobilisation stress in rats, *PDE7A* is upregulated via the stress response [65]. *CA8* is a carbonic anhydrase-related protein, expressed in the Purkinje cells and controls dendritic growth [66]. It has been found to affect anticonvulsant response during a seizure test in mice [67]. Mutations in *CA8* can cause ataxia [68,69] and mild mental retardation [70] *VPS26* plays a role in sensitisation to pain [71] diabetes susceptibility [72] and is involved in trafficking and sorting of transmembrane proteins [73].

In this study, we identify a total of five candidate genes for tonic immobility using a large-scale combined QTL/eQTL approach. Of these genes, *PRDX4* and *ACOT9* have by far the strongest support, having both strong correlations and the highest NEO causality scores. These represent the first actual candidate genes identified for tonic immobility in any vertebrate, despite this behaviour being remarkably widespread in a large number of species and a trait that has been under investigation since the 1600s. We find that tonic immobility shows no phenotypic correlation with inverted restraint behaviour; however, both *PRDX4* and *ACOT9* are also strong candidates for sociality/anxiety behaviour in the chicken. This evidence helps elucidate the role that tonic immobility has with anxiety in general and points to at least a partially shared genetic architecture between these traits. From a production perspective, the shackling behaviour appears to be independent to tonic immobility, at least in this intercross. However, this behaviour was strongly repeatable and the ability to map the genetic architecture of shackling behaviour to attempt to reduce this behaviour in domestic broiler stocks could be highly beneficial. Given the total production of chicken meat worldwide is around 90 million tons, and food waste due to poor pH can be as high as 48% [21,22,23], the saving in terms of environmental benefits, sustainability and welfare on a global scale represents a huge potential impact. The gene *PRDX4* has previously been shown to affect muscle pH [25]; however, this appears to be by a different mechanism than that controlling tonic immobility behaviour. Further studies are required to ascertain the relative mechanisms involved in both these traits. 

## Figures and Tables

**Table 1:** Tonic Immobility (TI) quantitative trait loci (QTL) identified in the main mapping population. Chromosome, position (in cM and chr:bp), additive and dominance effects, lower and upper confidence intervals (CI), as well as covariates used and any epistatic interactions incorporated are provided, as is the total variance explained by each QTL (*r*-squared) and the nearest genetic marker to the upper and lower C.I. Traits are maximum time spent in tonic immobility (TI maximum d.), average time spent in tonic immobility (TI average d.), time spent in tonic immobility in trial 1 (TI duration 1) and time spent in tonic immobility in trial 2 (TI duration 2).

Trait	Chr	Position	LOD	Add ± S.E.	Dom ±S.E.	Lower CI	Upper CI	Lower Marker	Position (Chr:bp)	Upper Marker	Position (Chr:bp)	Covariates	Interaction	*R* ^2^
TI maximum d.	1	2014	5.8	−19.9 ± 21.6	−25.7± 42.1	1991	2038	1_190334672	1:190334672	1_195271649	1:195271649	sex, batch, PC2,3,10	1@2014.0:2@485.0	3.7
TI duration 2	1	1750	7.3	−30.8 ± 11.7	−2.0 ± 15.4	1735	1756	Gg_rs10728648	1:144081810	snp-23-342-18608-S-2	1:151495059	sex, batch, PC2,6	1@1748.0:7@3.0	5
TI average d.	2	774	6.5	−10.8 ± 9.2	−28.7± 14	764	795	RBL1120	2:116617839	Gg_rs15146557	2:117659953	sex, batch, PC2,6	2@774.0:24@60.7	4.6
TI maximum d.	2	485	7.3	27.9 ± 13.1	−3.3 ± 17	474	495	Gg_rs14190959	2:60361704	Gg_rs15110213	2:65012153	sex, batch, PC2,3,10	1@2014.0:2@485.0	4.7
TI maximum d.	2	775	7.3	−9.9 ± 12.8	−18.1± 19.3	767	798	RBL1120	2:116617839	Gg_rs15146557	2:117659953	sex, batch, PC2,3,10	2@775.0:24@61.0	4.7
TI duration 2	2	181	7.9	−63.0 ± 14.9	−35.7± 18.3	170	195	Gg_rs15070042	2:19473457	2_23979784	2:23979784	sex, batch, PC2,6	24@60.7:2@181.0	5.2
TI duration 1	4	283	9.7	33.4 ± 10.5	11.4 ± 16.2	272	292	Gg_rs14446625	4:31963411	4_37860292	4:37860292	sex, batch, PC1,4	4@283.0:15@187.9	7.8
TI duration 1	6	259	6.9	5.6 ± 9.1	10.7 ± 12.2	247	270	6_25762392	6:25762392	Gg_rs14592224	6:30433595	sex, batch, PC1,4	6@258.7:10@185.0	5.5
TI duration 2	7	4	11.5	11.2 ± 13.5	48.5 ± 17.7	0	8	Gg_rs15826188	7:1654910	Gg_rs15828492	7:2444843	sex, batch, PC2,6	1@1748.0:7@3.0, 7@3.0:24@60.7	7.8
TI average d.	10	99	5.9	−15.1 ± 7.7	14.9 ± 10.1	86	109	Gg_rs14941656	10:2678686	Gg_rs14003134	10:5805005	sex, batch, PC2,6	10@99.0:20@247.7	4.1
TI maximum d.	10	139	8.4	−24 ± 11.4	−35.5 ± 15.1	133	144	10_9525779	10:9525779	Gg_rs14947769	10:11621480	sex, batch, PC2,3,10	10@139.0:12@85.0	5.4
TI duration 1	10	185	8.5	2.4 ± 9.5	−34.6± 12.2	176	198	Gg_rs14008254	10:12705455	GG_rs14951592	10:15605204	sex, batch, PC1,4	6@258.7:10@185.0	6.8
TI maximum d.	12	85	7.2	−24.2 ± 11.5	8.7 ± 14.7	77	190	Gg_rs13609494	12:5140626	12_14051161	12:14051161	sex, batch, PC2,3,10	10@139.0:12@85.0	4.7
TI duration 1	15	188	7.9	−18.0 ± 9.6	−19.5± 12.3	177	189	Gg_rs14095161	15:10467522	Gg_rs14095923	15:11490734	sex, batch, PC1,4	4@283.0:15@187.9	6.3
TI average d.	20	247.7	6.4	−9.4 ± 7.1	24 ± 10.1	237	252	Gg_rs15177950	20:10931547	Gg_rs14280872	20:13367853	sex, batch, PC2,6	10@99.0:20@247.7	4.8
TI average d.	24	60.7	7	−20.9 ± 8.5	−21.0± 11	53	67	GG_rs16194400	24:1782119	Gg_rs14294768	24:3003659	sex, batch, PC2,6	2@774.0:24@60.7	4.9
TI maximum d.	24	61	8.7	−26.6 ± 11.9	−25.0 ± 15.4	54	66	GG_rs16194400	24:1782119	Gg_rs14294768	24:3003659	sex, batch, PC2,3,10	2@775.0:24@61.0	5.6
TI duration 2	24	61	16.5	21.5 ± 15.3	−40.9 ± 18.7	54	65	GG_rs16194400	24:1782119	Gg_rs14294768	24:3003659	sex, batch, PC2,6	7@3.0:24@60.7, 24@60.7:2@181.0	11.5

**Table 2:** Causality analysis of the candidate genes in the eQTL sub-population of the F_8_ mapping population. Gene and probe name (in the microarray) are provided, as are the eQTL chromosomal location and position in cM. The *p*-value of the correlation with the behavioural trait, as well as which behavioural trait is also included. The QTL effect of the behaviour specifically in the eQTL sub-population is included, to allow the behavioural QTL strength of effect in this smaller sub-population to be assessed (* *p* < 0.05, ** *p* < 0.01, *** *p* < 0.001). Where two values are given the first is the significance of the behavioural QTL with just the single marker effect, the second is with the two marker epistatic interaction detected in the full sample included. Causality metrics leo.nb.oca and leo.nb.cpa measures are used to assess the strength of causality, with leo.nb.cpa values used when eQTL and QTL share the same genetic marker and leo.nb.oca values are used when eQTL and behavioural QTL have separate markers.

Gene	Probe Name	eQTL Chr	eQTL Position	eQTL LOD	Behaviour Trait	Behavioural Trait Correlation	Behavioural Trait *t*-Value	Behaviour QTL LOD	Behaviour QTL chr	Behaviour QTL Position	LEO.NB.OCA	LEO.NB.CPA	NEO Model *p*-Value
*ACOT9 probe 1*	ENSGALT00000026377_Q5F3B4_CHICK	10	138	8.6***	TI maximum d.	0.004	2.9	0.8/5.9***	10	139	-	0.50	0.88
*ACOT9 probe 1*	ENSGALT00000026377_Q5F3B4_CHICK	10	138	8.6***	TI maximum d.	0.004	2.9	1.5*/6.6***	12	85	0.12	0.50	0.99
*ACOT9 probe 2*	ENSGALT00000036634_Q5F3B4_CHICK	10	128	7.1***	TI maximum d.	0.02	2.3	0.8/5.9***	10	139	-	0.55	0.98
*ACOT9 probe 2*	ENSGALT00000036634_Q5F3B4_CHICK	10	128	7.1***	TI maximum d.	0.02	2.3	1.5*/6.6***	12	85	0.15	0.55	0.94
*CA8 probe 1*	603865613F1	2	778	6.7***	TI average d.	0.017	−2.4	1.4*	2	774	-	0.49	0.38
*CA8probe 1*	603865613F1	2	778	6.7***	TI maximum d.	0.02	−2.4	2.2**	2	775	-	0.31	0.20
*CA8 probe 2*	603863179F1	2	784	6.1***	TI average d.	0.03	−2.1	1.4*	2	774	0.05	0.03	<0.001
*CA8 probe 2*	603863179F1	2	784	6.1***	TI maximum d.	0.03	−2.1	2.2**	2	775	-	−0.30	<0.001
*PDE7A*	ENSGALT00000025015_LOC771318	2	784	6.6***	TI average d.	0.03	2.1	1.4*	2	774	0.04	0.31	<0.001
*PDE7A*	ENSGALT00000025015_LOC771318	2	784	6.6***	TI maximum d.	0.03	2.1	2.2**	2	775	-	−0.38	<0.001
*PRDX4*	ENSGALT00000026387_PRDX4	10	138.7	8.5***	TI maximum d.	0.0002	3.9	0.8/5.9***	10	139	-	0.38	0.66
*PRDX4*	ENSGALT00000026387_PRDX4	10	138.7	8.5***	TI maximum d.	0.0002	3.9	1.5*/6.6***	12	85	0.11	0.38	0.96
*VPS26*	603867427F1	24	67	6.6***	TI duration 2	0.004	−3.0	1.0 N.S.	24	61	-	-	-

**Table 3:** Partial Spearman’s correlations (controlling for sex) for tonic immobility, inverted restraint and muscle pH in the F_13_ verification cohort. Numbers above the diagonal show the *p*-value significance of each pairwise correlation (*p < 0.05, **p < 0.01, ***p < 0.001), whilst numbers below the diagonal are the Rho correlation coefficient.

Combined Data (Male and Female)		Initial Restraint Test		Pre-Slaughter Restraint		Tonic Immobility				pH	
		Duration	No. Flaps	Duration	No. Flaps	Duration 1	Duration 2	Average d.	Maximum d.	Initial	>5h pH
Initial restraint test	duration	-	<0.001***	0.33	0.53	0.38	0.58	0.82	0.74	0.39	0.40
	no. flaps	0.98	-	0.43	0.70	0.27	0.74	0.66	0.59	0.43	0.46
Pre-slaughter restraint	duration	0.15	0.12	-	<0.001***	0.60	0.85	0.84	0.77	0.87	0.21
	no. flaps	0.10	0.06	0.94	-	0.96	0.95	0.78	0.45	0.98	0.43
Tonic immobility	duration 1	0.14	0.17	0.08	0.01	-	<0.005**	<0.001***	<0.001***	0.96	0.68
	duration 2	−0.09	−0.05	0.03	−0.01	0.43	-	<0.001***	<0.001***	0.56	0.72
	average d.	0.04	0.07	0.03	−0.04	0.83	0.83	-	<0.001***	0.77	0.68
	maximum d.	0.05	0.09	−0.05	−0.12	0.81	0.74	0.95	-	0.70	0.87
pH	initial	0.13	0.12	0.03	0.00	−0.01	0.09	0.05	0.06	-	<0.005**
	>5h pH	0.13	0.11	0.19	0.12	0.06	0.06	0.06	0.03	0.42	-

## Supplementary Materials

The following are available online at https://www.mdpi.com/2073-4425/10/5/341/s1, Table S1: Descriptive statistics for the behavioural traits in the F_8_ mapping population. Table S2: Correlations between different tonic immobility measures in the F_8_ generation. Numbers above the diagonal show the p-value significance of each pairwise correlation (* = *p* < 0.05, ** *p* < 0.01, *** *p* < 0.001), whilst numbers below the diagonal are the correlation coefficient. Table S3. Full NEO output for the causality analysis.
