# Supplementary material for: Genetical Genomics of Tonic Immobility in the Chicken
Source: Genes (Basel). 2019 May 7;10(5):341. doi: 10.3390/genes10050341 (PMC6562468; doi:10.3390/genes10050341)
Supplement: Supplementary file 1 [file genes-10-00341-s001.pdf]

Supplementary information

Supplementary table 1. Descriptive statistics for the tonic immobility behavioural traits in the F_8 mapping population.

behaviour trait	mean (s)	median (s)	quartile range
TI duration 1	156	114	60–198
TI duration 2	168	109	57–218
TI maximum d.	222	165	104–210
TI average d.	161	125	78–221

Supplementary table 2. Correlations between different tonic immobility measures in the F8 generation. Numbers above the diagonal show the p-value significance of each pairwise correlation (* = P<0.05, ** P<0.01, ***P<0.001), whilst numbers below the diagonal are the correlation coefficient.

Correlation F8	TI duration 1	TI duration 2	TI maximum d.	TI average d.
TI duration 1	-	1×10^{-9}	$< 2 \times 10^{-16}$	2×10^{-16}
TI duration 2	0.26***	-	$< 2 \times 10^{-16}$	2×10^{-16}
TI maximum d.	0.69***	0.81***	-	2×10^{-16}
TI average d.	0.76***	0.83	0.95***	-

Supplementary table 3. Full NEO output for the causality analysis.

edge	LEO.NB.OCA	LEO.NB.CPA	Model.P.value.AtoB	LEO.NB.MAX	#_markers	trait1	trait2
trait1 -> trait2	0.548	0.548	0.975	0.548	1	ENSGALT00000036634_Q5F3B4_CHICK	max_TI
trait2 -> trait1	−7.11	NA	7.53×10^{-8}	−0.548	1	ENSGALT00000036634_Q5F3B4_CHICK	max_TI
trait1 -> trait2	0.148	0.548	0.939	0.148	2	ENSGALT00000036634_Q5F3B4_CHICK	max_TI
trait2 -> trait1	−0.263	−0.279	0.512	−0.263	2	ENSGALT00000036634_Q5F3B4_CHICK	max_TI
trait1 -> trait2	0.502	0.502	0.879	0.502	1	ENSGALT00000026377_Q5F3B4_CHICK	max_TI
trait2 -> trait1	−6.14	NA	6.44×10^{-7}	−0.502	1	ENSGALT00000026377_Q5F3B4_CHICK	max_TI
trait1 -> trait2	0.121	0.502	0.995	0.121	2	ENSGALT00000026377_Q5F3B4_CHICK	max_TI
trait2 -> trait1	−0.183	−0.261	0.653	−0.183	2	ENSGALT00000026377_Q5F3B4_CHICK	max_TI
trait1 -> trait2	0.312	0.312	0.204	0.312	1	X603865613F1	max_TI
trait2 -> trait1	−6.22	NA	1.23×10^{-7}	−0.937	1	X603865613F1	max_TI
trait1 -> trait2	0.494	0.494	0.377	0.494	1	X603865613F1	average_TI
trait2 -> trait1	−6.9	NA	4.75×10^{-8}	−0.73	1	X603865613F1	average_TI
trait1 -> trait2	0.0478	0.0341	1.63×10^{-78}	0.0341	2	X603863179F1	average_TI
trait2 -> trait1	−0.0478	−6.85	1.46×10^{-78}	−0.69	2	X603863179F1	average_TI
trait2 -> trait1	−6.71	NA	4.21×10^{-8}	−1.21	1	X603863179F1	max_TI
trait1 -> trait2	−0.303	−0.303	0.107	−0.303	1	X603863179F1	max_TI
trait1 -> trait2	0.0442	0.311	1.46×10^{-78}	0.311	2	ENSGALT00000025015_LOC771318	average_TI
trait2 -> trait1	−0.0442	−5.27	1.32×10^{-78}	−0.698	2	ENSGALT00000025015_LOC771318	average_TI
trait1 -> trait2	0.0604	−0.381	1.23×10^{-79}	−0.381	2	ENSGALT00000025015_LOC771318	max_TI
trait2 -> trait1	−0.0604	−5.18	1.07×10^{-79}	−1.2	2	ENSGALT00000025015_LOC771318	max_TI
trait1 -> trait2	0.377	0.377	0.658	0.377	1	ENSGALT00000026387_PRDX4	max_TI
trait2 -> trait1	−5.45	NA	2.33×10^{-6}	−0.377	1	ENSGALT00000026387_PRDX4	max_TI
